# Large-scale photonic inverse design: computational challenges and breakthroughs

**DOI:** 10.1515/nanoph-2024-0127

**Published:** 2024-06-07

**Authors:** Chanik Kang, Chaejin Park, Myunghoo Lee, Joonho Kang, Min Seok Jang, Haejun Chung

**Affiliations:** 26716 Hanyang University, Seoul, South Korea; Korea Advanced Institute of Science & Technology, Daejeon, South Korea

**Keywords:** large-scale, inverse design, computational challenges

## Abstract

Recent advancements in inverse design approaches, exemplified by their large-scale optimization of all geometrical degrees of freedom, have provided a significant paradigm shift in photonic design. However, these innovative strategies still require full-wave Maxwell solutions to compute the gradients concerning the desired figure of merit, imposing, prohibitive computational demands on conventional computing platforms. This review analyzes the computational challenges associated with the design of large-scale photonic structures. It delves into the adequacy of various electromagnetic solvers for large-scale designs, from conventional to neural network-based solvers, and discusses their suitability and limitations. Furthermore, this review evaluates the research on optimization techniques, analyzes their advantages and disadvantages in large-scale applications, and sheds light on cutting-edge studies that combine neural networks with inverse design for large-scale applications. Through this comprehensive examination, this review aims to provide insights into navigating the landscape of large-scale design and advocate for strategic advancements in optimization methods, solver selection, and the integration of neural networks to overcome computational barriers, thereby guiding future advancements in large-scale photonic design.

## 1 Introduction

**Figure 1:**
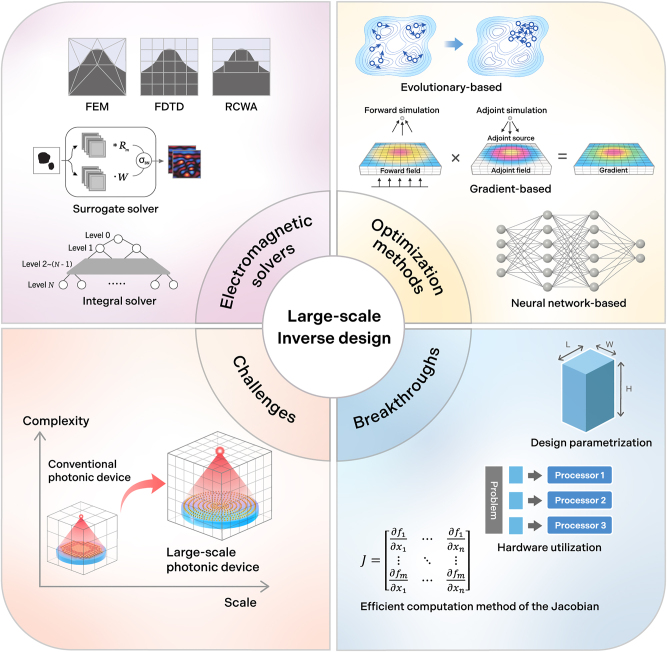
Overview of large-scale inverse design [16]. Reprinted with permission from ACS Photonics Copyright 2023 American Chemical Society [17], licensed under CC BY 4.0.

Over the past two decades, nanophotonics has contributed to the advancement of both fundamental science and industrial technology through the development of photonic devices that can produce desired outcomes, such as scattering and polarization. This achievement was realized through the manipulation of light–matter interactions at the sub-wavelength scale, and has enabled a shift from mere theoretical exploration to the practical application of these technologies. Noteworthy applications have emerged, including the enhancement of virtual- and augmented-reality technologies [1], [2], holographic imaging systems [3]–[5], light detection and ranging (LiDAR) [6], and the development of metalenses [7]–[9]. These applications are notable owing to their adept manipulation of light scattering and resonance to shape the desired wavefront, leveraging metasurfaces comprising meta-atoms engineered in alignment with Huygens’ principle [10]. The strategic assembly of these sub-wavelength scatterers into coherent structures has demonstrated superior efficacy over traditional optical solutions across various fields, such as the production of high-numerical-aperture (NA) lenses [11] and advancement of holography and silicon photonic chips [12], [13]. In addition, pre-optimized meta-atoms, representing different phases and amplitudes are reused in supercells, enabling this approach to be highly efficient in large-scale designs. However, the application of this approach encounters challenges in the creation of devices capable of multifunctional or broadband operation, particularly because of the difficulty in designing meta-atoms that can support rapidly changing wavefronts across the spatial and frequency domains [14], [15] (Figure 1).

Inverse design introduces a paradigm transition to address the limitations of metasurface designs that rely on meta-atoms. This approach, which is markedly distinct from conventional forward design methodologies, seeks to address physical challenges through the application of new mathematical frameworks. It necessitates defining the functionality of a device in terms of an objective function and engaging in an iterative optimization process. Initial inverse design studies utilized gradient-free schemes, including genetic algorithms [18] and particle swarm optimization [19], which are categorized as evolutionary algorithms. However, thousands of evaluations are typically required to discover an optimal solution, rendering them impractical for solving large-scale problems. Subsequently, gradient-based optimization, known as adjoint optimization [20], [21] or topology optimization [22]–[25], has been introduced to solve problems with high degrees of freedom (DoF), such as beam demultiplexers [26], [27], achromatic metalenses [28], [29], beamsplitter [30], and nonlinear optical modulators [31]. Additionally, the field of meta-optics has expanded beyond two-dimensional to encompass three-dimensional meta-optics [32], which offers a higher degree of freedom. For example, 3D meta-optics metalens [33] and beamsplitter [34] employed an ‘objective-first’ algorithm, which prioritizes achieving performance objectives in the field configurations before conforming to Maxwell’s equations [26]. These methods offer large-scale computational optimization for various photonic problems. However, discovering optimal designs typically requires more than a hundred iterations of full-scale simulations [35], [36]. Many photonic-design challenges include large simulation sizes exceeding a few hundred thousand *λ*; therefore, gradient-based optimization still faces critical obstacles in solving large-scale design problems.

In response to the computational challenges of designing large-scale, high-efficiency photonic devices, a comprehensive strategy incorporating hardware acceleration, deep-learning techniques, and algorithmic innovations has been proposed. Hardware acceleration, markedly reduces simulation time primarily through the emergence of high-performance graphics processing units (GPUs) and specialized processors, fostering more efficient design exploration and optimization. Concurrently, deep learning models are promising candidates for approximating the solution of Maxwell’s equations with less computational overhead [37]. In addition, pre-trained deep learning models offer highly efficient photonic designs without additional simulations [36], [38]. In summary, the recent progress in new design approaches efficiently addresses the complex design space of nanophotonic devices, marking a pivotal evolution in design tactics to enhance performance and scalability, and unlock new possibilities for designing large-scale and intricate devices.

This review discusses the challenges in designing large-area devices and, examines how current research has overcome these obstacles through numerical computation and deep neural networks (DNNs). Furthermore, it explores emerging research on GPU-based design techniques and compares it with conventional methods, offering insights into future research directions.

## 2 Computational challenges in large-scale simulations

The limitations of computer memory capacity impose constraints on the scale at which structures can be designed and simulated in computational environments. This constraint significantly affects the maximum feasible size of simulations. Performing operations that exceed the RAM capacity in von Neumann architecture [39] significantly slows the process, thereby restricting the scale of the simulation. Furthermore, the computational throughput of processing units significantly affects the temporal efficiency of simulations, thereby influencing the rate at which the simulations can be executed.

For example, designing a large-scale photonic structure, such as a 50 μm^2^ metasurface with a 5 nm mesh size using the finite-difference time-domain (FDTD) method, may require approximately 100 h of simulation time and 100 GB of memory consumption [40]. Although these methodologies are adept at delineating the complexities inherent in nanoscale physics, their scalability is hampered by computational and memory constraints. Xue et al. [17] observed that the practical upper limit for the diameter of inverse-designed, fully three-dimensional metasurfaces is approximately 200*λ*, correlating to approximately 100 μm^2^ in terms of visible light wavelengths in three dimensions. Moreover, the time required to run the FDTD simulation was proportional to the domain size, assuming that the resolution remained constant. Consequently, simulating and designing a full 1 cm^2^ region at the same resolution (5 nm mesh size) would require approximately 20,000 h (equivalent to 2.28 years) and 20,000 GB of memory (equivalent to 20 TB), which implies that a total of 190 petabytes of computing space are ultimately required. These figures highlight the formidable barriers encountered in processing large-scale simulations.

Even in a hypothetical scenario of unlimited memory space, the computational performance can still encounter bottlenecks owing to the memory transfer bandwidth of the architecture of contemporary computing systems. This architecture encompasses both dynamic random-access memory (DRAM) and cache memory, with the latter serving as an immediate storage solution for data frequently accessed by computational units. However, the bandwidth of the cache memory [41], [42], which is crucial for transferring data to processing units, is limited and cannot be expanded indefinitely. This limitation can lead to situations in which processing units are unable to receive data promptly despite ample memory capacity. This may result in bottlenecks that affect performance, regardless of the total memory size. This problem is exacerbated during a “cache miss” [43], where the data is not available in the cache memory, necessitating retrieval from the slower, larger DRAM and imposing a performance penalty. According to Lu et al. [44], despite the capability of each thread to perform approximately 10^9^ floating-point operations per second, all input and output values must be stored in a register bank with a limited capacity of approximately 1 KB. This requirement underscores the challenges posed by limited memory bandwidth and storage capacity, highlighting the critical need for efficient data management and architecture optimization in high-performance computing applications.

This issue becomes particularly noticeable during a cache miss, when the data are not readily available in the cache memory. This necessitates retrieving data from the slower, larger DRAM, thereby imposing a performance detriment. Because all data and instructions must pass through the memory to reach the central processing unit (CPU), memory performance is also crucial for delivering data and instructions to the CPU. However, the slow pace of improvement in memory performance, which is only an approximately 7 % increase compared to the 60 % increase in CPU performance, represents another issue in modern computing. This disparity implies that the capabilities of processing units may not be able to fully utilized [45], [46].

This disparity, encapsulated in the term “memory wall,” [47] poses an increasingly significant challenge as computational requirements increase. In the contemporary computation landscape, substantial memory capacities cannot obviate the intrinsic limitations posed by the cache memory bandwidth, which can severely restrict the speed of memory transfers. This limitation becomes especially evident in simulations that exhaust the available memory capacity, thereby diminishing the computational efficiency as the system experiences data-management challenges.

In response to these limitations, recent studies have focused on harnessing GPU technology to bolster computational speeds. CPUs have evolved to enhance performance through single-core efficiency, While CPUs have evolved so that their performance is enhanced through single-core efficiency; however GPUs have leveraged their extensive core architecture to enhance performance, as shown in Table 1. Recent trends also indicated a convergence in the cost-effectiveness of GPUs relative to CPUs for identical specific computational tasks, marking a significant shift from the previously higher expense associated with GPU utilization.

**Table 1:** Comparison between CPU and GPU. In the following, we compare CPUs and GPUs in terms of memory, computational capabilities, and the relative cost of performing identical operations. This comparative analysis highlights the differences between the two in handling computing tasks and provides a clear understanding of their respective efficiencies and applications.

Feature	CPU (AMD 7995WX)	GPU (NVIDIA H200)
# Of cores	96	More than 10,000
Memory capacity	Over 1 TB (DDR5, 8-channel)	80 GB (GDDR)
fp64 computation power (TFLOPs)	6	35
Price	1	Approximately 4
Price per core	1	0.039
Cost for 1 TFLOPs of computation	0.167	0.114

However, the architecture of GPU memory presents a challenge. In contrast to CPUs that typically employ DRAM, GPUs use graphics DDR (GDDR) memory, which is characterized by higher transfer speeds and a larger bandwidth. Despite these benefits, the GPU memory remains a persistent challenge. GDDR memory is typically more expensive than standard DDR memory, reflecting its specialized design and performance capabilities, which can affect the overall cost of devices and systems. Therefore, the overall cost of GPU-accelerated computing is unfeasible for large-scale computations.

Therefore, regardless of whether CPUs or GPUs are used, the key to facilitating large-scale simulations for the design of large-scale photonic device centers is to minimize the simulation complexity. This requires the development and application of strategies that efficiently construct and simulate spaces while optimizing their structures. At its core, this involves choosing appropriate computational solvers for Maxwell’s equations and adopting suitable optimization methods, such as inverse design methodology, which are critical for achieving effective and efficient design processes.

## 3 Computational electromagnetic solvers for large-scale inverse design

Photonics, the science of light manipulation, fundamentally relies on Maxwell’s equations to describe light–matter interactions. The difficulty in deriving analytical solutions for most electromagnetic (EM) problems necessitates the adoption of numerical methods as alternatives to approximate the solutions. This reliance on numerical approximations has not only become common in photonics but has also marked a significant shift in the approach to complex EM challenges.

The advent of numerical methods has historically revolutionized the manner in which mathematical analysis has been conducted across various engineering domains. Before the era of modern computing, analytical methods were predominantly used, and the complexity of studies was constrained to avoid intricate calculations [48–56]. The transition to a computational paradigm, driven by advancements in computing technology, has dramatically expanded our ability to address high-order numerical problems with unprecedented efficiency and performance. This progress is also demonstrated by EM simulation tools, which are essential for designing and investigating photonic systems, offering a cost-effective alternative in both time and resources compared with physical experimentation.

The leap in computing power and algorithmic sophistication has fundamentally altered the landscape of numerical methods. Modern computing technologies enable efficient navigation through extensive calculations, thereby addressing complex numerical problems across a diverse range of scientific domains. Recent investments in computing resources and the development of algorithmic techniques have accelerated this trend, significantly improving the overall computational effectiveness and productivity of methods. The advent of such computational capabilities has not only simplified the modeling process for intricate photonic structures but also broadened the scope of EM research. This evolution underscores the critical role of modern computing in enhancing our ability to simulate, understand, and innovate within the photonic landscape and beyond; thereby setting a new benchmark for what is achievable in scientific exploration and technological advancement.

In the field of photonics, the emergence of subwavelength-scale structures, along with advancements in fabrication techniques, has ignited an intense pursuit of identifying optimal device designs from a broad spectrum of design possibilities. Two approaches may be used to achieve this: forward and inverse design. Forward design involves assembling small, well-understood components to create a larger device, as observed in metasurfaces, where meta-atoms are combined using a “unit-cell approach” [57], [58]. This method relies on EM simulations to catalog the optical behaviors of basic shapes.

Conversely, inverse design seeks to discover a device structure that delivers a specific figure of merit (FoM), framing the search as an optimization problem across a design parameter space with complexity varying according to the topology of the device. For simpler devices such as photonic crystals [59], diffraction gratings [60], and nano-antennas [61], [62], designers often select a fixed topology based on physical intuition and then employ parameter sweeps to find an optimal configuration. However, the fixed nature of these designs may result in limited performance of the device. Adopting a freeform approach allows for a broader exploration of design possibilities, albeit at the cost of increased computational demands for optimization, whether through gradient-based or machine-learning methods [63], [64].

As the EM problem expands across spatiotemporal scales, the number of unit elements dividing the simulation volume, such as spatial cells, meshes, and time steps, increases. Managing these increasing computations within a reasonable running time and available memory space is essential. Consequently, the appropriate choice of the EM simulation method, which significantly influences computational complexity, is critical, particularly for large-scale problems.

This section delves into computational EM simulation methods suitable for large-scale problems, starting with an overview of Maxwell’s equations and their boundary conditions. We explore commonly used computational methods, including the finite element method (FEM) [65], FDTD [66], finite-difference frequency-domain (FDFD) [67], and rigorous coupled-wave analysis (RCWA) [68]–[70], along with recent advancements in machine learning-based surrogate solvers that offer promising alternatives to traditional EM simulations.

### 3.1 Maxwell’s equations and boundary conditions

Maxwell’s equations [71] stand as the foundational pillars of EM field theory. These equations articulate the behavior of EM fields and encapsulate the principles of electromagnetism into four critical equations: Ampere’s law, Faraday’s law, Gauss’s law for electricity, and Gauss’s law for magnetism, as identified in Equations (1)–(4). This concise formulation provides a mathematical framework for understanding and predicting interactions between electric and magnetic fields in various physical contexts. In these equations, *H* denotes the magnetic field, *J* the current density, *D* the electric displacement, *E* the electric field, *B* the magnetic flux density, *ρ* the electric charge density, and *t* represents time.

(1) $$\nabla \times H=J+\frac{\partial D}{\partial t}$$

(2) $$\nabla \times E=-\frac{\partial B}{\partial t}$$

(3) ∇⋅D=ρ

(4) ∇⋅B=0

Furthermore, the relationship between the magnetic field and electric displacement can be expressed through $H=\frac{B}{\mu_{0}}-M$ and *D* = *ϵ*_0_ *E* + *P*, where *M* and *P* denote the magnetization and polarization, respectively. The constants *μ*_0_ and *ϵ*_0_ are the magnetic permeability and electric permittivity in free space, respectively.

To uniquely determine the electric- and magnetic-field solutions in a given scenario, boundary conditions must be applied in conjunction with the differential equations. For example, at the boundary separating two distinct media characterized by their respective magnetic permeability and electric permittivity values $\left(\mu_{1},\epsilon_{1}\right)$ and $\left(\mu_{2},\epsilon_{2}\right)$ , the EM fields must satisfy certain continuity conditions. These conditions, which stem directly from Maxwell’s equations, ensure that the behavior of EM fields is correctly modeled even at the interface between different materials.

The boundary condition $\hat{n}\cdot \left(B_{2}-B_{1}\right)=0$ ensures the continuity of the magnetic flux density’s normal component across the interface between two media. However, $\hat{n}\cdot \left(D_{2}-D_{1}\right)=\rho_{s}$ reflects a discontinuity in the normal component of the electric field at the interface, where *ρ*_*s*_ represents the surface charge density.

Special consideration is necessary when the medium exhibits unique physical properties. For example, the internal electric field is nullified inside a perfect electric conductor. Another notable scenario involves a perfectly matched layer [72], which is an absorbing boundary condition designed to simulate open-region problems by effectively truncating the computational domain such that it absorbs outgoing waves, thereby preventing reflections that can affect the accuracy of the simulation.

### 3.2 Conventional EM solvers

Building on our exploration of Maxwell’s equations and their associated boundary conditions, we now advance to the discretization of EM fields. This critical juncture allows us to represent these fields numerically by confining their infinite DoFs to a manageable discrete set. This approach involves assigning values to a discrete collection of oriented submanifolds, effectively characterizing the field across the following dimensions: cells (3D), faces (2D), edges (1D), and vertices (0D). However, not all collections of these submanifolds are suitable for discretization because the integral form of Maxwell’s equations (Equations (1)–(4)) necessitates careful consideration of surface boundaries within a bounded domain. The arrangement of these submanifolds in a structured network, or mesh, is essential. In practice, triangular meshes are often employed for two-dimensional domains, whereas tetrahedral meshes are preferred for three-dimensional spaces, owing to their flexibility and comprehensive coverage.

A commonly adopted method for discretizing Maxwell’s equations in their differential form is the finite difference (FD) method [73], which approximates the derivatives as the differences between the field values at adjacent grid points. Two prominent EM simulation methods that use FD are the FDTD and FDFD methods. These methods are tailored for time and frequency domain analyses, respectively, with FDTD especially valued for its straightforward implementation in uniform Cartesian grids. Despite its advantages, FDTD is not without its drawbacks, notably a “staircase” approximation issue [74] when modeling complex boundaries. This challenge that has been extensively analyzed and documented, including by Cangellaris and Wright.

To address such limitations, unstructured grid-based methods such as FEM are employed, offering superior flexibility particularly in dealing with complex geometries. The adaptability of FEM spans a wide array of applications not limited to EM theory and is also crucial in domains such as structural mechanics [75], fluid dynamics [76], heat transfer [77], and mass transport [78].

The time needed for FDTD simulations increases linearly as the domain expands while maintaining a constant resolution. However, to address phase accumulation errors caused by finite-difference sampling in larger scattering regions, the resolution must be increased, leading to significantly longer computation times, particularly for large scattering scenarios. Integral solvers tackle this issue by directly solving integral equations instead of differentials [17]. Techniques such as discrete dipole approximation and method of moments are used to discretize the integral form of Maxwell’s equations, providing alternative solutions to handle the computational challenges in EM simulations. Additionally, for EM scattering from periodic structures, the RCWA offers a semi-analytical solution by leveraging the Bloch wave expansion.

The following subsections provide an in-depth review of some of these fundamental EM simulation techniques, including FEM, FDTD, and RCWA, and provide insights into their operational mechanics and areas of most effective applications, as summarized in Table 2.

**Table 2:** The summary, pros, and cons of various core numerical tools for EM simulation.

	FEM	FDTD	RCWA
Key idea	Unstructured meshing, and solving matrix equations	Iterative leapfrog time-stepping	Solving Bloch eigenmodes
Response domain	Space-domain	Time-domain	Space-domain
Grid	Unstructured grid	Structured grid	Structured grid
Accuracy	Very accurate	Depends on mesh refinement	Depends on the Fourier orders
Periodic	Both applicable to periodic and non-periodic	Both applicable to periodic and non-periodic	Applicable for periodic
Pros	High resolution for complex geometries	Easy implementation, versatile, and low memory consumption	Adept for periodic structures
Cons	Requires large computing resources (both CPU time and memory)	Difficult to handle oblique boundaries, and slow for small devices	Needs high Fourier order to converge
Good for	Steady-state, fine geometry problems	Transient response, and large-scale problems	Layered media, scattering problems
Maximum simulation domain reported^a^	32.2*λ* × 47.6*λ* [79]	170*λ* × 100*λ* × 53*λ* [80]	20,000*λ* × 20,000*λ* [40]

^a^Maximum simulation domains are written in electrical dimensions.

#### 3.2.1 Finite element method

FEM traces its origins back to the 1940s and was initially developed as a numerical technique for solving complex problems in structural engineering, particularly civil engineering [75] and aeronautics [81]. An early example of its application is found in the work of Levy [81] on the structural analysis of delta airplane wings. This period marks the advent of the FEM, which is characterized by its novel strategy of subdividing a large problem into smaller, manageable units, termed finite elements, to simplify intricate calculations. This methodological innovation was motivated by the demand for more precise tools for structural analysis, particularly in aircraft and aerospace engineering, which catalyzed its widespread adoption and evolution into a multifaceted tool utilized across diverse scientific and engineering fields [82].

**Figure 2:**
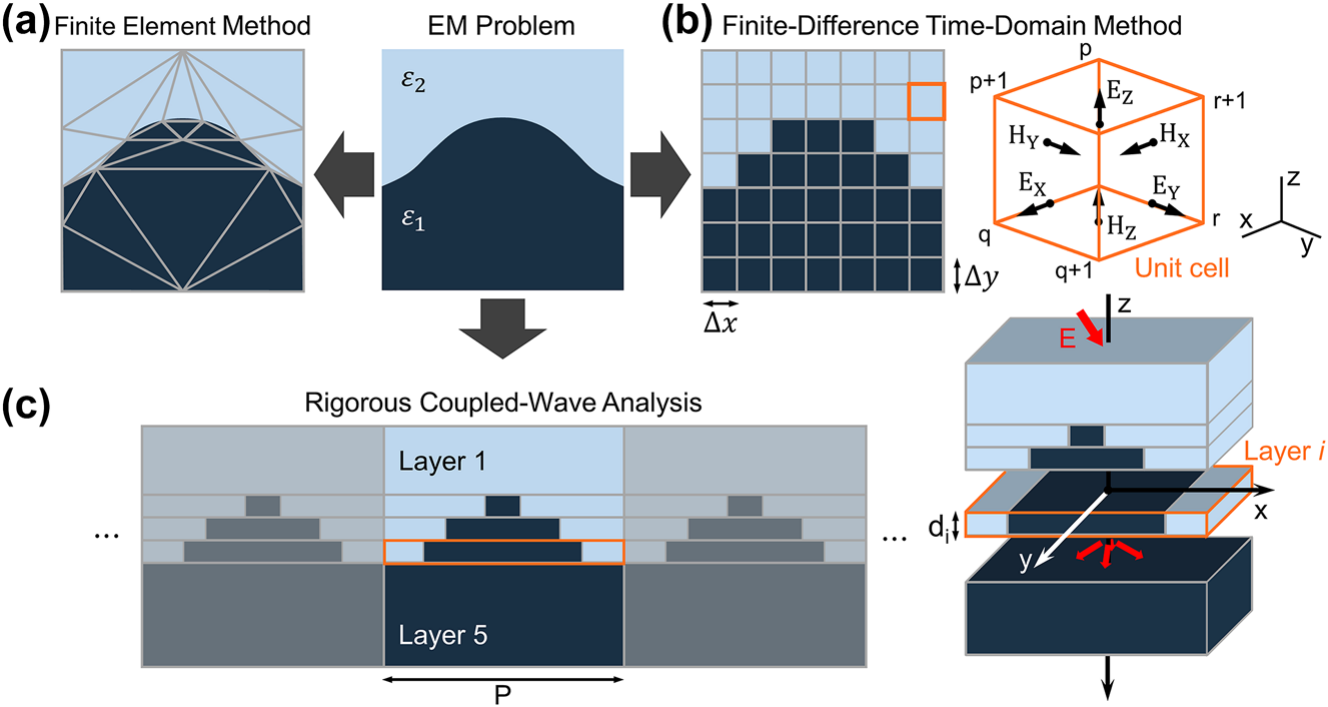
Schematic of computational electromagnetic techniques. Large-scale photonic design problems may require large-area electromagnetic simulations, highlighting the importance of choosing an appropriate simulation method. Schematic of (a) FEM, (b) FDTD, and (c) RCWA.

The core of the FEM involves dividing the domain of the solution into smaller, simpler entities called elements through meshing, as illustrated in Figure 2a. These elements, which take forms such as triangles, quadrilaterals, tetrahedra, prisms, and hexahedra, enable the construction of unstructured meshes that are adept at capturing complex geometries. After meshing, the solution is approximated through a finite set of basis functions, typically low-order polynomials that are non-zero over only a limited span of adjoining elements. Central to FEM is the application of the Galerkin method [83], which aims to eliminate the weighted residuals of the differential equation. This is achieved by utilizing test or weighting functions, often identical to the basis functions, to minimize the residual in a weak formulation approach.

**Figure 3:**
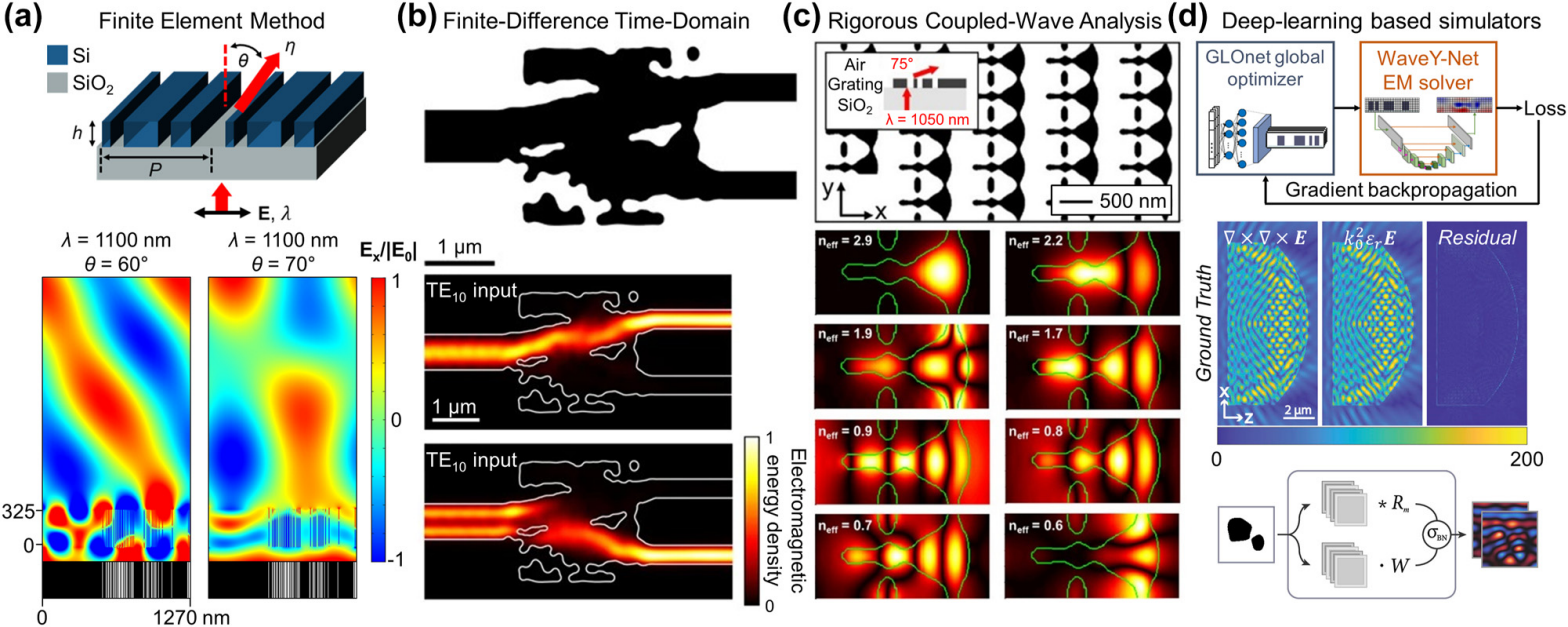
Numerical simulation methods for a variety of photonic design problems. (a) A silicon-based metagrating functioning as a one-dimensional beam deflector (a, top) that deflects TM-polarized light at a wavelength of 1100 nm and *E* field profiles (a, bottom) of the optimized device, calculated using FEM simulations. Figures adapted with permission from Park et al. [84]. Licensed under CC BY 4.0. (b) A silicon-based spatial-mode demultiplexer that routes TE10 and TE20 modes to the TE10 mode (b, top) and its electromagnetic energy density profiles (b, bottom) calculated using FDTD simulations. Figures adapted with permission from Piggott, A.Y., Petykiewicz, J., Su, L. et al. [85]. Licensed under CC BY 4.0. (c) Schematic of the metagrating as a two-dimensional beam deflector (c, top) for TE-and TM-polarized incidence wave at a wavelength of 1050 nm and *H* field profiles corresponding to each mode simulated from RCWA (c, bottom). Figures adapted with permission from Ref. [86]. Copyright 2017, American Chemical Society. (d) Deep-learning-based simulators including the wave Y-Net, a surrogate simulator for periodic structures using a physics-augmented deep neural network (DNN) (d, top), MaxwellNet, a DNN where the residual of Maxwell’s equations are used as the physics-driven loss (d, middle), and a Fourier neural operator (FNO), a surrogate solver for scattering problems (d, bottom). Figure (d, top) adapted with permission from Ref. [87]. Copyright 2022, American Chemical Society. Figure (d, middle) adapted with permission from Ref. [88]. Licensed under CC BY 4.0. Figure (d, bottom) adapted with permission from Ref. [16]. Copyright 2023, American Chemical Society.

A notable advantage of the FEM is its compatibility with unstructured meshes, providing unparalleled flexibility in modeling complex geometries and facilitating localized refinement. This is crucial for accurately capturing fine details or areas with rapid changes in permittivity and permeability at boundaries. For example, an *E*-field profile for an inverse-designed silicon metagrating with a minimum feature size of 5 nm, as depicted in Figure 3a, exemplifies the capability of FEM to accurately model the effects of minuscule structures. Figure 3a highlights the precision of the method in representing complex physical behaviors, demonstrating how the *E*-field in an optimized device under one set of conditions can differ markedly from that under another, underscoring the usefulness of the FEM in capturing nuanced phenomena.

The FEM was used to actively address industrial problems on a large scale during the 1990s, focusing on large-scale fluid-structure interactions. A key strategy for addressing these challenges was the application of the FEM in conjunction with parallel computing resources. Early adaptations of FEM for moving boundary problems in structural mechanics utilized parallel processors for intensive computations [89]. Another attempt was made in the heat conduction problem, where specific functionalities of the FEM were replaced by neural networks (NNs) to perform computations with parallel processors [77]. Moreover, for EM problems, some studies implemented parallel solvers for FEM formulations to model large devices, successfully addressing simple EM problems [79], [90], [91].

Generally, large-scale EM problems pose a challenge for the FEM, often demanding substantial computational resources, including CPU time and memory. However a unique and efficient computing management solution is hardly proposed. This is because of the necessity of solving a linear system of equations in time-domain simulations, a task that is more resource-intensive than methods such as FDTD, which can directly update fields using explicit formulas. Despite these challenges, the versatility and accuracy of the FEM have cemented its status as a method of choice in numerous engineering and scientific endeavors. Continuous advancements has further enhanced its efficiency and broadened its applicability. Prominent commercial platforms that utilize the FEM include ANSYS and COMSOL Multiphysics, which offer advanced tools for a wide range of applications in electromagnetics and beyond.

#### 3.2.2 Finite-difference time-domain method

FDTD is a pivotal computational electromagnetics technique for solving Maxwell’s equations. As illustrated in Figure 2b, FDTD employs a staggered grid, known as Yee’s grid [92], for the spatial discretization of EM fields. It strategically positions the electric fields at the edges of a cell and the magnetic fields at the center, efficiently streamlining the computation of Maxwell’s curl equations.

Central-difference approximations are utilized in FDTD to calculate both the temporal and spatial derivatives in Maxwell’s equations. The method divides the simulation domain into a lattice-like grid for spatial considerations and time into discrete intervals for temporal analysis. This division supports an explicit time-stepping algorithm that alternates updates between electric and magnetic fields – magnetic fields at half-time steps, and electric fields at full-time steps – in a leapfrogged sequence. Such a staggered updating sequence not only ensures numerical stability but also preserves the natural coupling between the electric and magnetic fields.

FDTD is lauded for its precision in modeling complex geometries and materials across a wide frequency range, coupled with its straightforward implementation. It particularly excels in time-domain responses, such as in analyzing transient or broadband signals [93]. The memory efficiency of FDTD, which eliminates the need for matrix storage, is another advantage. However, FDTD requires careful spatial discretization to depict wave phenomena precisely, which can substantially increase the computational burden of large-scale or high-frequency applications. Numerical dispersion and stability, dependent on grid resolution and timestep magnitude, are limitations that necessitate adherence to the Courant–Friedrichs–Lewy condition [94] to ensure stable and accurate simulations by balancing timestep sizes with grid dimensions.

Parallel to FDTD, the FDFD method provides an alternative to Maxwell’s equations, particularly for the frequency response analyses of EM fields. Unlike FDTD, FDFD discretizes fields in space while keeping the time domain continuous, facilitating straightforward field distribution analyses at specific frequencies. This approach is efficient for examining the resonant behavior or filtering characteristics of photonic structures.

To address large-scale EM challenges, FDTD and FDFD methods have emerged as leading computational EM techniques, offering significant advantages over alternative approaches. Although they have significant computational demands owing to their volumetric characteristics, they boast massive parallelizability compared with the FEM. Unlike the FEM, FDTD and FDFD are exempt from solving a linear system problem, which has the potential to fully harness the benefits of the computing system. Their computational complexity, including both memory and CPU time scales, varies linearly with the size of the EM problem, whereas those of the FEM depend on the expected accuracy of the solution [95], which is typically more complex. Some studies have presented practical examples of large-scale simulations using FDTD [96]–[98].

The adaptability and broad applicability of FDTD establish it as a fundamental tool in computational electromagnetics. Its utility is enhanced by open-source software such as Meep, which offers full scriptability and memory parallelism, and Ceviche, which supports both FDTD and FDFD along with automatic differentiation (AD). Commercial platforms such as ANSYS Lumerical provide a robust set of tools for industries that require rapid virtual prototyping and detailed verification. An exemplary application, illustrated in Figure 3b, features an EM energy density plot for a silicon demultiplexer device analyzed through FDTD, demonstrating the effectiveness of the method in addressing real-world engineering challenges.

#### 3.2.3 Rigorous coupled-wave analysis

RCWA, also referred to as the Fourier modal method [99], is a refined technique designed to examine light interactions within periodic structures. RCWA offers a unique perspective for analyzing periodic domains by breaking them down into a series of uniform vertical layers. These layers are characterized by horizontal variations in material distribution, as illustrated in Figure 2c.

By applying Bloch’s theorem, which states that the periodic nature of a structure confines the electric field solutions to a discrete set, RCWA calculates the Bloch modes within the diffraction layers using the Bloch eigenmode solver [100]. These modes, expressed through Fourier components, are intimately connected to the material geometry of each layer, which are depicted in Fourier space. This method then leverages an enhanced transfer matrix method [70] to ascertain the EM field propagation across the structure, enabling precise calculations of the light dynamics within the system.

RCWA is particularly adept at analyzing 2D and 3D periodic structures, including diffraction gratings [101], photonic crystals [102], and resonant waveguides [103]. Its notable applications include the simulation of two-dimensional silicon metagratings, where RCWA facilitates a detailed study of the *H* field profile and potential modes within the structure, as shown in Figure 3c. Unlike iterative methods such as FDTD, FDFD, and FEM, RCWA adopts a direct strategy that substantially minimizes computational demands. The computing speed is particularly beneficial when dealing with large-scale periodic structures. However, its efficiency and accuracy are contingent on the selection of the Fourier components. While increasing these components can improve the simulation precision, it simultaneously increases the requirements for computational power and matrix storage capacity, presenting a balance between detail and resource allocation.

Continuous improvements aim to optimize the convergence rates of the RCWA, striving for simulations that are both accurate and resource-efficient. The leading open-source tools for RCWA are RETICOLO [104] and S4 [105], developed for MATLAB and Python environments, respectively. Newer software, such as MAXIM [106], introduces user-friendly graphical interfaces. Moreover, enhancements in convergence and AD are featured in Meent [107], marking significant strides in making RCWA more accessible and powerful for photonic research.

### 3.3 Neural network-based surrogate solvers for electromagnetic computations

The integration of NNs into computational electromagnetics significantly enhances the analysis and understanding of EM phenomena. The foundational work on artificial NNs (ANNs) in 1989 demonstrated their capability to approximate any function [108], laying the groundwork for leveraging NNs in this field. By processing inputs through multiple hidden layers and utilizing backpropagation coupled with AD to optimize the loss function, NNs play a crucial role in enhancing the modeling of light interactions with complex structures. This progress has led to the development of NN-based surrogate solvers, which aim to dramatically streamline the simulation process by offering significantly reduced inference times and errors, positioning them as potentially superior alternatives to traditional EM simulation techniques.

Initial studies underscored the potential of tandem NNs as surrogate solvers in electromagnetics, particularly for predicting optical properties [86], [109]–[115]. Recent advances have explored complex NN architectures including convolutional neural networks (CNNs) and graph neural networks (GNNs) to model intricate optical phenomena. CNNs, which are renowned for their efficacy in image recognition through convolution and pooling operations, have been applied in photonics to predict vector fields. The U-Net architecture [116], recognized for its balance between model expressiveness and data efficiency, has been particularly effective in predicting the internal fields within silicon nanostructures, as demonstrated by Wiecha et al. [117].

As shown in Figure 3d (top), Chen et al. introduced WaveY-Net, a U-Net-based framework, to predict field distributions in dielectric nanophotonic structures using only magnetic field data for training and deriving electric fields using Maxwell’s equations [87]. This method not only enhances model accuracy but also aligns predictions closely with physical principles.

A primary challenge in training NNs is generating sufficient input-output pairs, either experimentally or via conventional EM simulators. To mitigate this, recent approaches have incorporated physical laws directly into NN training, thereby reducing reliance on large datasets. For example, as shown in Figure 3d (middle), Lim et al. employed the residuals of Maxwell’s equations as a physics-based loss function for training MaxwellNet, effectively reducing the requirement for larger datasets [88]. Furthermore, Kang et al. introduced an optical data augmentation algorithm integrated with adjoint sensitivity analysis, which augmented input-output paired data more than 300 times even with enhanced device efficiency [118].

GNNs have gained attention for their versatility in addressing EM problems, particularly their capacity to process data represented by nodes and edges, regardless of their size and connectivity. They have also been applied to photonics, specifically for simulating interactions between light and structures. In a notable study by Khoram et al., trained GNNs proved effective in solving EM scattering problems for metasurfaces of any size [119]. Furthermore, Kuhn et al. adapted the FDTD propagation scheme within GNNs, enabling the prediction of electric field evolution over a fixed timestep in unfamiliar scenarios based on the initial field distribution [120].

Operator networks, particularly the Fourier neural operator (FNO), have revolutionized computational modeling by learning the entire family of mappings between function spaces instead of single functions [121], [122]. Li et al. demonstrated the capability of an FNO to approximate any continuous operator by demonstrating its efficiency in addressing the partial differential equations common in photonics [123]. Furthermore, Augenstein et al. illustrated the superiority of FNO over traditional FDTD methods in EM scattering problems, demonstrating enhanced accuracy and expressiveness with fewer parameters, as shown in Figure 3d (bottom) [16].

As deep learning continues to advance, optimizing CNNs, managing GNNs for large-scale graphs, and employing FNOs for high-mode operations have become central challenges. Overcoming these limitations, along with hardware acceleration strategies, is essential for scaling up to large-scale problems. This discussion sets the stage for the concluding section of Section 3, which delves into strategies to address these challenges in greater detail.

### 3.4 Hardware acceleration and parallel computing

**Figure 4:**
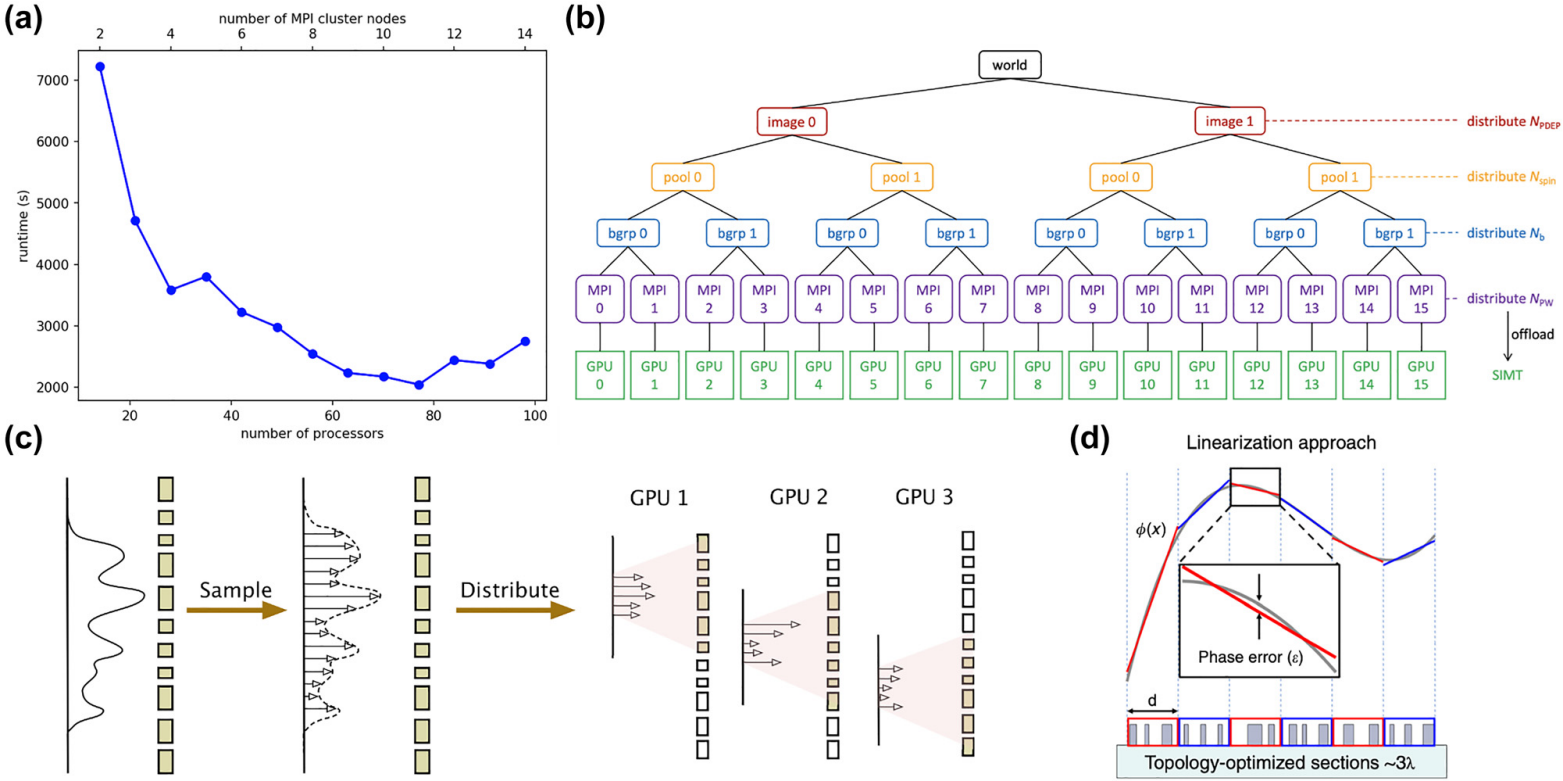
Examples of hardware-aware solutions used in photonics design. (a) Simulation time can be changed depending on the number of processors. A single problem can be solved by parallelizing through MPI across multiple processors. This parallelization can reduce runtime effectively [128]. Licensed under GPL-2.0. (b) Example of multilevel parallelization for the case of 16 total MPI processes [129]. Reprinted with permission from J. Chem. Theory Comput. Copyright 2022 American Chemical Society. (c) Schematic of the simulation distribution method. The incidence field is initially sampled and expressed as a superposition of $\vec{J}$ inc sources. Subsequently, individual GPUs simulate smaller sets of $\vec{J}$ inc sources and the nearby metasurface [130]. Licensed under CC BY 4.0. (d) The desired phase profile is partitioned and then linearized for metagrating designs. Then, topology optimization is applied to optimize toward a linearized phase profile [23]. Licensed under CC BY 4.0.

The previous sections on conventional and surrogate EM solvers highlighted their advantages and limitations. However, a common challenge identified across all the solver types is the substantial demand for simulation time and memory, particularly for large-scale applications. This section discusses hardware-aware solutions for large-scale EM simulations, including parallel computing and GPU acceleration. Parallel computing illustrated in Figure 4a, a technique that distributes complex problems across multiple processors for simultaneous execution, has been widely used in contemporary computing since its inception. This method is particularly effective for analyzing nanophotonic structures that require extensive simulations. For example, designing a 50 μm^2^ metasurface using the FDTD method would necessitate approximately 100 h and 100 GB of memory [40]. However, parallel computing across a multi-core CPU can significantly accelerate this process by distributing the workload among the cores. Similarly, the acceleration of solving Maxwell’s equations via computer architecture employs specialized hardware, such as a GPU, digital signal processor (DSP) [124], and field-programmable gate array (FPGA) [125]. Commercial EM analysis tools, such as Lumerical and COMSOL, have integrated parallelization features, simplifying the implementation process. In the field of open-source tools, Meep incorporates message passing interface (MPI)-based parallel programming techniques which described in Figure 4b alongside load balancers that allocate core numbers based on the problem size, facilitating efficient parallel computing [126]. In addition, efforts are being made to enable researchers to create large-scale designs without using high-performance computers. The photonic device research and development (R&D) tool PlanOpSim [127] supports cloud computing and can perform computations over a large area of 6.3 × 6.3 mm^2^, amounting to 169,000,000 meta-atoms. PlanOpSim significantly reduces the barriers for researchers and developers engaging in advanced photonic device R&D by leveraging the power of cloud computing to handle the computational demands of large-scale simulations and designs.

Recent advancements in GPU-accelerated computing techniques have led to significant developments in EM solvers. Among these, the GPU-accelerated FDTD solver, ‘Tidy3d’ [131], [132] has demonstrated remarkable computing speeds. This solver can execute simulations of large-area metalenses with turnaround times on the scale of minutes, representing a substantial improvement in computational efficiency. Specifically, 2.09 billion grid cells with 64,275 time steps can be computed in approximately 3 min, which would take approximately 27 h on 96 processors in a traditional CPU-based FDTD simulation. Furthermore, ‘Tidy3d’ has been utilized for the simulation of 3D metalenses of unprecedented size, showcasing its potential for advancing optical simulation and design.

Lu et al. [44] developed the open-source tool ‘fdtd-z’, which leverages CUDA and a systolic update scheme to adapt the FDTD update algorithm for the massively parallel architecture of GPUs. This method efficiently manages data transfers within the GPU memory hierarchy, which is crucial for sparse computations such as those encountered in nanophotonic simulations.

**Figure 5:**
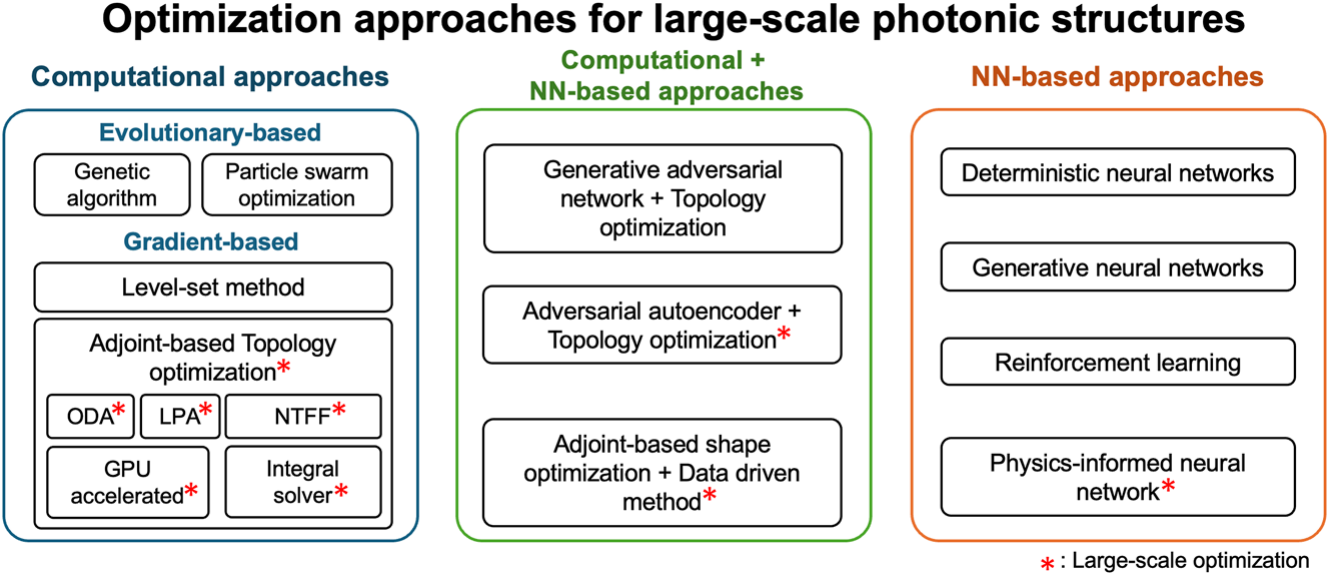
Overview of optimization methodologies for large-scale photonic structure including evolutionary-based, gradient-based, and integrated neural network-based. Asterisks (*) indicate favorable approaches for large-scale inverse design compared to other inverse design methods.

Parallel computing divides complex problems among multiple processors and addresses computational speed delays due to memory speed in information sharing among hardware. Skarda et al. [130] introduced a low-overhead distribution strategy for simulating and optimizing large-area metasurfaces, as illustrated in Figure 4c, notably reducing the computational speed delays by incorporating hardware characteristics. Their approach, which utilized a GPU-based implementation of the transition-matrix method, enabled efficient simulation and adjoint sensitivity analysis of large-area metasurfaces, significantly improving the simulation time with a scalable number of computing nodes. This strategy facilitated distributed simulation over areas exceeding 600*λ* × 600*λ*, accurately accounting for scatterer interactions beyond the locally periodic approximation (Figure 5).

## 4 Large-scale optimization method

For an effective large-scale inverse design, efficient and precise forward simulations are imperative, as highlighted in Section 3. However, the iterative nature of optimization, coupled with significant time requirements, poses substantial challenges in optimizing nanophotonic devices with high DoFs (e.g., more than 10^6^). Approaching such expansive design tasks necessitates addressing the computational memory limitations encountered during both simulation and iterative optimization phases. In general, solving partial differential equations using numerical analysis techniques requires storage to save and prompt calculated variables. The size of the storage system typically scales with the size of the problem, requiring a huge memory capacity in large-scale problems. Furthermore, optimization algorithms that iteratively run full-scale Maxwell simulations require computational resources to determine an optimal structure that maximizes the FoM or minimizes the loss function. Notably, some inverse design methodologies, such as adjoint optimization, require gradient calculations for the designable space. This iterative procedure requires significant memory and time. Therefore, reducing the computational complexity has emerged as a critical endeavor in the optimization of large-scale nanophotonic devices. To reduce complexity, choosing an appropriate optimization method tailored to the specific problem of interest is crucial. In this section, we focus on the computational obstacles of each inverse design methodology and the key approaches that have led to breakthroughs.

### 4.1 Evolutionary-based approaches

Evolutionary-based approaches have been used in the initial stages of nanophotonic inverse design, combining intuitive structural designs with computational algorithms such as evolutionary algorithms (EA). EAs are population-based metaheuristic algorithms that mimic the natural selection process by iteratively applying genetic operations such as recombination and mutation [133]–[135]. They aimed to identify the viable solutions in resource-constrained environments by evaluating and comparing the fitness values of successive generations. The most prominent EAs used in the design of nanophotonic devices are genetic algorithms (GAs) and particle swarm optimization (PSO). In this section, we provide an analysis of how GA and PSO are employed in nanophotonics research and discuss the challenges associated with their application in large-scale inverse design.

#### 4.1.1 Genetic algorithm

Inspired by natural selection and evolutionary principles, GA was introduced in the early 1970s by Holland [18]. It conceptualizes potential solutions as individuals within a population, with each solution represented by a chromosome comprising genes that encode the problem parameters. The GA involves three fundamental operations: selection, crossover, and mutation. Selection identifies the best chromosomes based on their performance on a defined objective function, referred to as the fitness function. During the crossover, the selected chromosomes exchange their gene sequences to produce new offspring, incorporating the characteristics of the parent chromosomes. Finally, the mutation stage introduces random gene variations into the offspring chromosomes, which enables the algorithm to explore a wider range of solutions and avoid being trapped in local optima.

**Figure 6:**
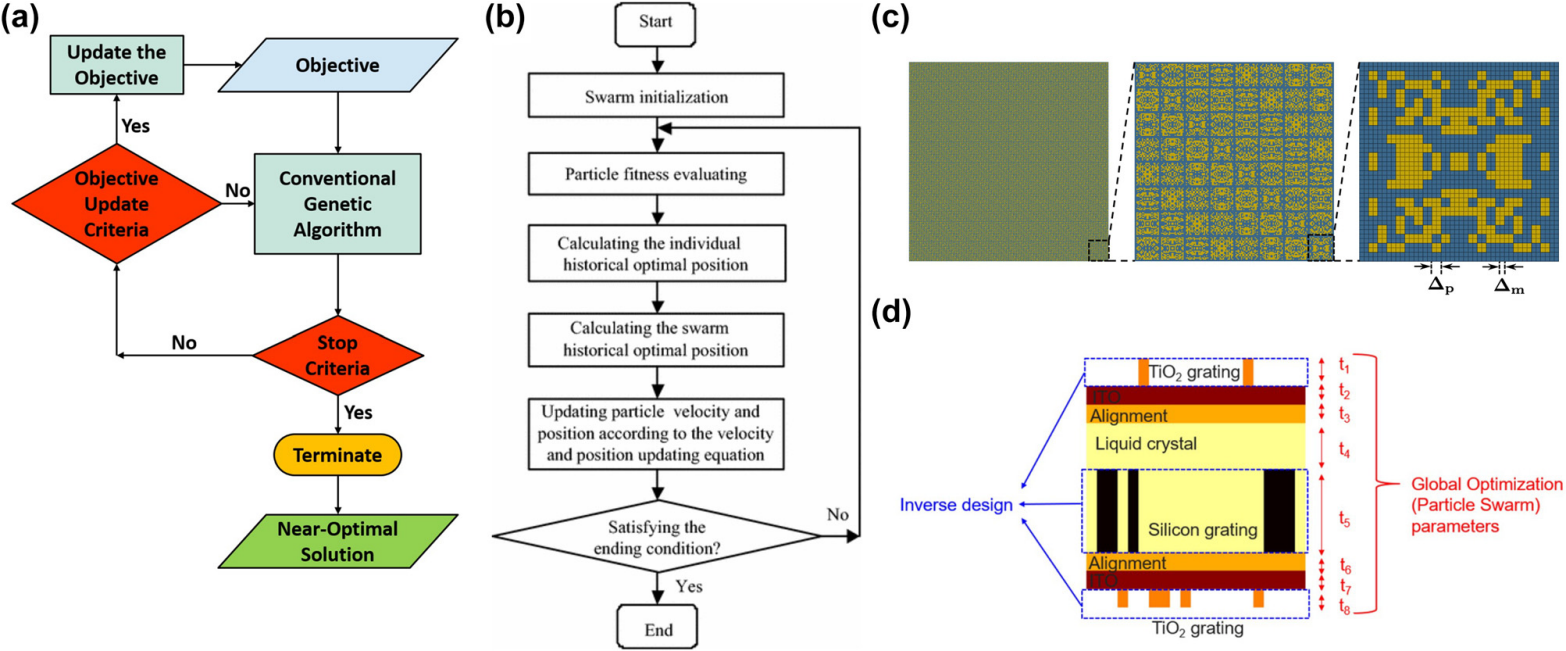
Evolutionary-based optimizations in photonics. (a) Flowchart illustrating the adaptive genetic algorithm (AGA) for designing optical metasurfaces, showcasing the iterative optimization process. Figures adapted with permission from Jafar-Zanjani, S., Inampudi, S., and Mosallaei, H. [141]. Licensed under CC BY 4.0. (b) Flowchart of the particle swarm optimization (PSO) algorithm. Copyright 2018, Wang, D., Tan, D. and Liu, L, under exclusive license to Springer-Verlag GmbH Germany, part of Springer Nature [142]. (c) Binary pattern representation of a designed metasurface with AGA for beam deflection to specific angles (*θ*_0_, *ϕ*_0_) = (30°, 45°), including an 8 × 8 super-cell and a detailed view of the lower right unit-cell with specified mesh and pixel sizes. Figures adapted with permission from Jafar-Zanjani, S., Inampudi, S., and Mosallaei, H. [141]. Licensed under CC BY 4.0. (d) Schematic diagram illustrating a combined strategy of PSO and adjoint-based inverse design for metasurfaces, highlighting the iterative procedure of each agent conducting an inverse design with variable layer thicknesses for optimized performance. Figures adapted with permission from Ref. [143]. Copyright 2020, American Chemical Society.

GAs are particularly effective in addressing problems with discrete solution domains, multidimensional function domains, and non-differentiable objective functions [136]. These strengths have facilitated their application in various nanophotonic device designs, including plasmonic metasurfaces [137], photonic crystals [138], metalenses [139], [140] and broadband absorbers [141]. Additionally, adaptive variations, such as the adaptive genetic algorithm (AGA), have been developed for multi-objective optimization, where the optimization criteria are dynamically adjusted according to the priority levels. This approach, illustrated in Figure 6a and c, was effectively applied to various photonic designs by Jafer et al. [141]. Unlike GAs, which use constant weights for all objectives throughout the optimization process, the AGA employs a dynamic approach in which the optimization criteria can be adjusted during the process based on the priorities of the objectives. This study demonstrates four AGA-assisted photonic designs: a plasmonic metasurface that steers the incident beam in the desired direction, dual-beam leaky-wave antenna, birefringent metasurface unit-cells, and an infrared emitting/absorbing visible-transparent metasurface.

However, GAs encounter challenges in large-scale device design because of their high computational cost and inefficiency [144]. The computational cost and inefficiency of GAs, exacerbated by the need for numerous function evaluations, render them less feasible for designs with a high DoF. The process becomes increasingly resource-intensive with the scale of the design variables, demanding careful tuning of the GA parameters, such as the mutation rates, to maintain efficiency.

#### 4.1.2 Particle swarm optimization

By contrast, PSO offers a different approach to global optimization, drawing inspiration from the social behaviors of fish schools and bird flocks. Introduced by Kennedy and Eberhart [19], this global optimization technique models a group of entities known as particles, each representing a potential solution within the search space. Every particle is characterized by distinct position and velocity attributes that are iteratively updated based on specific update rules. The essence of PSO lies in mimicking the collective search behavior observed in nature, aiming to pinpoint the optimal particle configuration in terms of position and velocity within the solution space. A general PSO operation initiates with particles dispersed randomly across the solution space. Subsequently, during each iteration, the particle velocities are adjusted based on collective information, directing their movements towards optimal positions in the next generation [142]. The search process completes when the swarm converges to an optimal solution or satisfies a predefined termination condition. A flowchart of the general PSO algorithm is shown in Figure 6b.

PSOs have been effectively implemented in the design of nanophotonic devices, leveraging their strengths in addressing non-differentiable and discrete functions. Their applications span a diverse range of structures, including photonic crystal waveguides [145], broadband absorbers [146], diffraction grating structures [147], and meta-grating beam deflectors [143]. A notable implementation by Forestiere et al. [148] demonstrated the capability of PSO to optimally arrange metal nanoparticles to enhance broadband plasmonic fields across the visible spectrum. In this study an array of 55 × 55 plasmonic nanospheres was designed to maximize a multi-objective function by utilizing the binary version of the PSO algorithm. The PSO algorithm has been extensively integrated with FDTD [149] and RCWA [150] for objective function evaluation. These studies underscore the utility of PSO in refining binary or grating-based nanostructures, attesting to the robustness and versatility of PSO for the optimization of intricate nanophotonic designs.

However, the design of large-scale nanostructures with high DoFs presents considerable obstacles, particularly owing to the constrained design space of binary or grating structures and the extensive time required for global optimization and iterative simulations. Evolutionary-based methods, while effective in nanophotonic inverse designs, tend to show the greatest efficacy at lower DoFs [144]. Therefore, these algorithms must develop strategies to overcome the limitations encountered in high DoF scenarios. A noteworthy strategy was presented by Chung and Miller [143], who illustrated the design of tunable devices using a combination of adjoint optimization techniques and PSO, as shown in Figure 6d. Adjoint optimization was employed to determine the optimal functional structures of TiO_2_ and silicon, whereas PSO was utilized to refine the layer thicknesses. This integrated approach mitigated the constraints of PSO related to DoFs and reduced the risk of converging on local minima, which is a common limitation in adjoint-based local optimization methods.

### 4.2 Gradient-based approaches

Gradient-free optimization methods, including evolutionary optimization, often encounter significant computational demands when addressing complex design challenges because of their high-dimensional nature [64]. In scenarios where computational resources are a limiting factor, gradient-based optimization techniques can provide a more efficient solution for large-scale applications. Consequently, gradient-based strategies, such as the adjoint method [151], often in conjunction with topology optimization [22], have yielded numerous successful outcomes [126], [152] in inverse design tasks.

The concept of topology optimization, particularly highlighted after Jensen and Sigmund’s groundbreaking introduction of its use in designing photonic crystal structures [153], [154], has since become a cornerstone in photonic device design, particularly for devices that require a high DoF. This method strategically segments the design space into computational elements or pixels, considering each as a distinct design parameter, thereby effectively increasing the DoFs of the system. In pursuit of superior device structures, the application of level-set and adjoint density-based topology optimization has been observed in studies conducted by Lalau-Keraly et al. [155], Borel et al. [156], Burger and Stanley [157], and Gerken and Miller [158]. These methodologies leveraged a gradient-based approach to pinpoint the locally optimal photonic configurations. The level-set method is notably adept at managing smaller design spaces, and density-based topology optimization has established a niche in high DoF applications [159].

However, density-based topology optimization faces two primary computational challenges in designing complex and multifunctional large-scale devices: the time and memory required for iterative EM simulations and the efficient computation of FoM gradients. The strategies used to address the first challenge are detailed in Section 4.

The current section details the approaches for addressing the second challenge and evaluates the comparative memory and speed complexity when these methods are utilized in FDTD simulations to optimize the photonic structures.

Gradient-based optimization updates the parameters (mostly the permittivity) of each pixel in the design space based on the FoM gradients of the pixels. The gradients of the FoM with respect to numerous design variables can be expressed as the Jacobian of a function **F** that maps the input parameters (*N*_input_) to the output properties (*N*_output_). If we assume that function **F**: $R^{N_{\text{input}}}\to R^{N_{\text{output}}}$ such that the first-order derivatives of each element exist on $R^{N_{\text{input}}}$ , the Jacobian matrix of **F** is defined as an *N*_output_ × *N*_input_ matrix:

(5) $$J=\left[\begin{array}{ccccc} \frac{\partial F_{1}}{\partial x_{1}} & \ldots & \frac{\partial F_{1}}{\partial x_{i}} & \ldots & \frac{\partial F_{1}}{\partial x_{N_{\text{input}}}}\\ \vdots & & \ddots & & \vdots \\ \frac{\partial F_{N_{\text{output}}}}{\partial x_{1}} & \ldots & \frac{\partial F_{N_{\text{output}}}}{\partial x_{i}} & \ldots & \frac{\partial F_{N_{\text{output}}}}{\partial x_{N_{\text{input}}}} \end{array}\right]$$

where $x\in R^{N_{\text{input}}}$ is a vector of input variables, and *x*_*i*_ is the *i*th input parameter.

As part of the inverse design process for computing the Jacobian, one can choose from a few different methods, including the finite-difference method, adjoint method [22], automatic differentiation [160], [161], and direct differentiation [162]. The finite-difference method is the most classical approach to calculating the gradient of a function, calculating the output changes of the function based on a given small change (Δ_*i*_) in each input variable. The finite-difference method approximates the gradient of **F** as follows:

(6) $$\frac{\text{d}F}{dx_{i}}\approx \frac{F\left(x+\Delta_{i}\hat{i}\right)-F\left(x\right)}{\Delta_{i}}$$

where $\hat{i}$ is a unit vector indicating the *i*th index. The finite-difference method, which calculates the gradients individually for each design variable, experiences a proportional increase in the total computation time as the number of input parameters increases. This method is particularly effective in inverse design problems characterized by a small number of input parameters relative to a larger number of outputs (*N*_input_ ≪ *N*_output_). However, in the context of designing large-scale devices in which the number of design variables is substantially large, this approach necessitates an extensive number of EM simulations. Furthermore, the computed gradients may be true for the specific step sizes (Δ_*i*_). These limitations significantly restrict the applicability of the finite-difference method for the optimization of large-scale devices.

#### 4.2.1 Adjoint-based method

**Figure 7:**
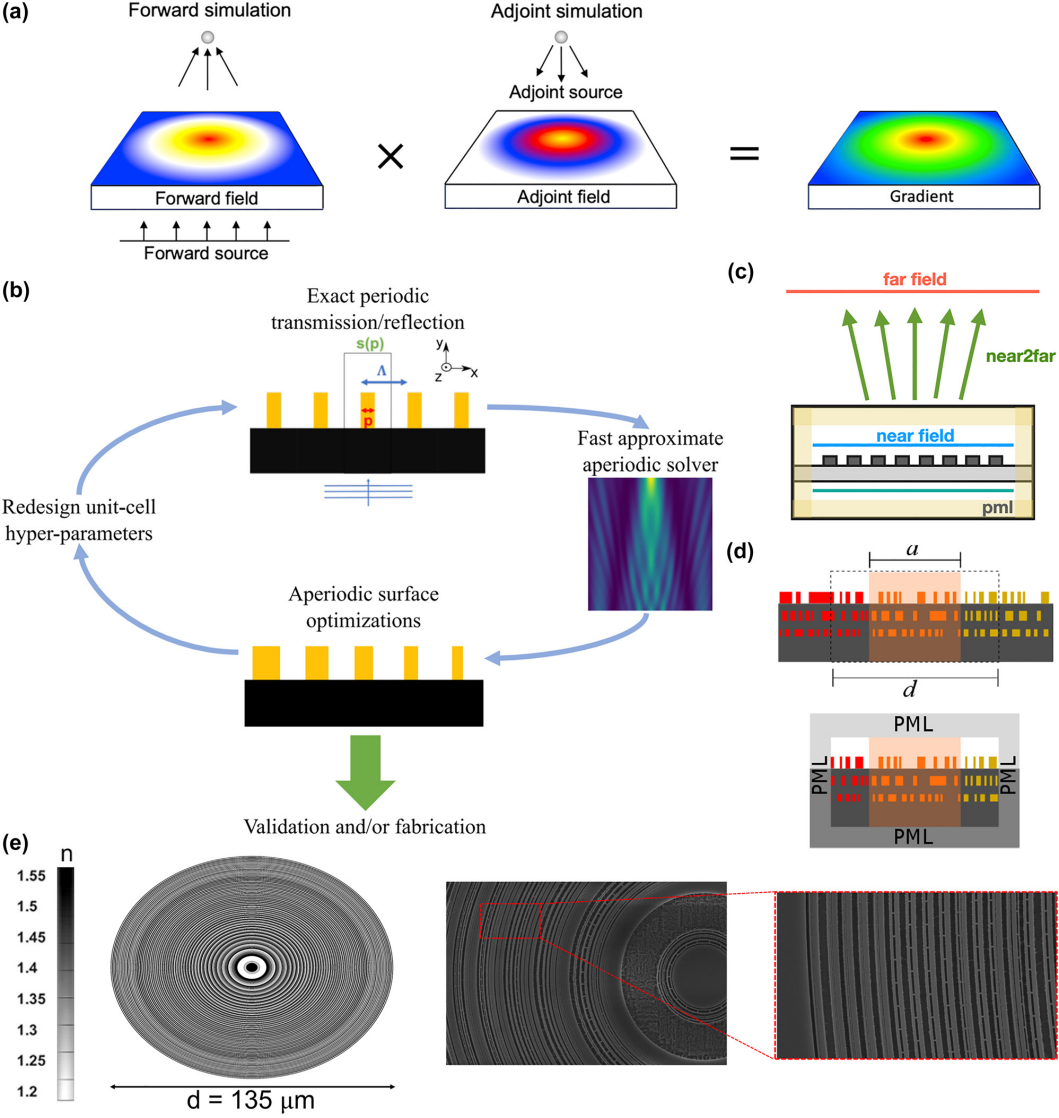
Adjoint-based approaches to large-scale inverse design in photonics. (a) Illustration of adjoint sensitivity analysis, leveraging forward and adjoint simulations to compute exact gradients with minimal simulations. Adapted with permission from Kang et al. [118]. Licensed under CC BY 4.0. (b) Schematic representation of a design method that utilizes exact Maxwell solutions for periodic unit cells to approximate solutions for aperiodic compositions, enabling the large-scale optimization of metasurface parameters. Adapted with permission from Pestourie et al. [165]. Licensed under CC BY 4.0. (c) Illustration of near-to-far-field transformation for designing metalenses, showcasing the designable region and computation of far-field transformations. Adapted with permission from Christiansen et al. [166]. Licensed under CC BY 4.0. (d) Schematic of the overlapping domain method for the optimization of aperiodic multi-layered meta-structures. Adapted with permission from Lin, and Johnson [167]. Licensed under CC BY 4.0. (e) Inverse-designed high-numerical-aperture metalens for maskless lithography. Adapted with permission from Chung et al. [7]. Licensed under CC BY 4.0.

Conversely, the adjoint method enables the computation of the Jacobian with only two simulations, a forward simulation and an adjoint simulation, irrespective of the number of design parameters. This method assesses the ‘forward’ EM responses of each pixel within the design domain via a forward simulation, and subsequently calculates the ‘adjoint’ responses by simulating with adjoint sources. The gradient of the FoM of each pixel is determined by the interaction of the “forward” and ‘adjoint’ EM fields in the frequency domain:

(7) $$\frac{\text{dFoM}}{\text{d}\epsilon_{i}}=\frac{1}{\pi }Re\left[\underset{\Delta_{\omega }}{\int }\omega^{2}E^{A}\left(x_{i},\omega \right)\cdot E^{*}\left(x_{i},\omega \right)\text{d}\omega \right]$$

where *e*_*i*_ is the permittivity of *i*th pixel, **E** ^*A*^(**x**_*i*_, *ω*) is the adjoint responses of the pixel, and E*(**x**_*i*_, *ω*) is a complex conjugate of its forward responses. The detailed mathematical derivation of this relationship has been well described by Tang et al. [162] and Hughes et al. [161], [163]. The gradient of a function using the adjoint method was derived by applying Lagrange multipliers, as shown by Chung et al. [164]. Miller [22] derived the gradient by exploiting the symmetry of Green’s function, which represents the Lorentz reciprocity between an oscillating current source and the fields induced by the source. Despite the slightly different notations depending on the derivation methodologies, the derived formulae indicate that the gradients of the FoM can be easily computed by dotting the forward field and adjoint field profiles of each pixel, as illustrated in Figure 7a. Based on the calculated Jacobian, the gradient descent optimization algorithm updates the permittivity of each pixel in each iteration. This iterative process continues until a local optimum is reached, which, although local, represents a satisfactory FoM.

As the adjoint method requires only one additional simulation for each output property, the method distinguishes itself with advantages in problems with a large number of input parameters (*N*_input_ ≫ *N*_output_). Given its advantages, the adjoint method has been widely adopted in photonic inverse design problems characterized by a small number of response features and large array of design parameters. A typical application is in a metalens, which requires one or two performance metrics at the focal point.

The utilization of the adjoint method in topology optimization has facilitated a high focusing efficiency, broadband operation, and high NA metalens. The demand for practical metalens applications, such as telescope [168] and lithography [7], has led to studies focused on the optimization of large-scale metalenses. A notable contribution by Pestourie et al. [165] showcased a metalens with a 361*λ* diameter, capable of functioning across the visible region. To solve the inverse design problem of a large area structure, they employed local periodic approximation (LPA) and near-to-far-field (NTFF) transformation to the adjoint method. LPA approximates the scattering field to the composition of a periodically divided scattering field, which is widely used in forward design problems such as unit-cell-based designs [169]. Because LPA discretizes the structure with periodic boundary conditions, its utilization effectively enhances the optimization of vast areas. They reduced the error from the approximation via interpolation using the Chebyshev methods [170], which constructs a polynomial approximated function. After obtaining the field using the LPA and Chebyshev methods, they used the NTFF transformation [66] to predict the EM field response at the target points. NTFF transformation regards the fields in the ‘near’ plane as equivalent current sources in accordance with the principle of equivalence [171], [172]. The ‘far’ fields at any points above the ‘near’ plane can be calculated with the equivalent current sources and Maxwell Green’s function. By skipping the simulation space between the ‘near’ plane and targeted ‘far’ points, NTFF transformation can significantly scale down the simulation space. Figure 7c shows the schematic of NTFF transformation. This study successfully implemented an adjoint-based large-scale inverse design in tandem with the LPA and NTFF transformation, as illustrated in Figure 7b.

LPA has demonstrated efficacy in the design of moderate-NA or narrow bandwidth operating metalenses. However, the approximation can break down into more complex and rapidly varying metasurfaces, such as broadband high-NA metalenses, resulting in non-negligible errors. Lin et al. [167] introduced topology optimization integrated with overlapping-domain approximation (ODA) instead of using LPA. ODA simulates a larger domain than the unit-cells because the LPA error comes from the assumption that the boundary of the unit-cells is connected with a Bloch boundary condition [173], [174]. As illustrated in Figure 7d, the ODA sets the simulation space to a size *d* that overlaps the neighboring cells, whose size is represented as *a* (*d* > *a*). The simulation domain is also padded by perfectly matched layers, which are absorbing boundaries. They demonstrated that ODA can improve the accuracy of unit-cell-based field approximation and designed high-NA (0.71) large-scale (200*λ*) broadband (480–700 nm) operating metalens.

Because inverse design methods utilizing LPA or ODA approximate the field distribution from discretized simulation results, a decline in functionality becomes inevitable when unexpected coupling occurs among the unit cells. To avoid these approximations, Chung et al. [7] designed and fabricated high-NA (0.67) large-scale (333.33*λ*) metalens through forward and adjoint simulations over the entire structure using cylindrical symmetry. They demonstrated that a full-wave Maxwell simulation, in this case, FDTD, and the adjoint method could scale up the design structure according to the increased number of CPU cores (64 cores).

#### 4.2.2 Automatic differentiation

In inverse design, the objective is to find a design configuration that maximizes a specific FoM. The adjoint method offers a powerful approach to optimizing devices with a vast number of parameters by numerically evaluating the exact Jacobian of the FoM in two simulations. Due to its high accuracy and low computational complexity, the adjoint method has been used in many photonic devices. However, in some cases, the desired FoM has a complicated form, leading to significant challenges in implementing adjoint source formulation. For example, when designing photonic crystals [59] or implementing spatial filters and threshold steps to satisfy the fabrication constraints [86], [175]–[177], it is challenging to calculate the gradient solely relying on the adjoint method.

**Figure 8:**
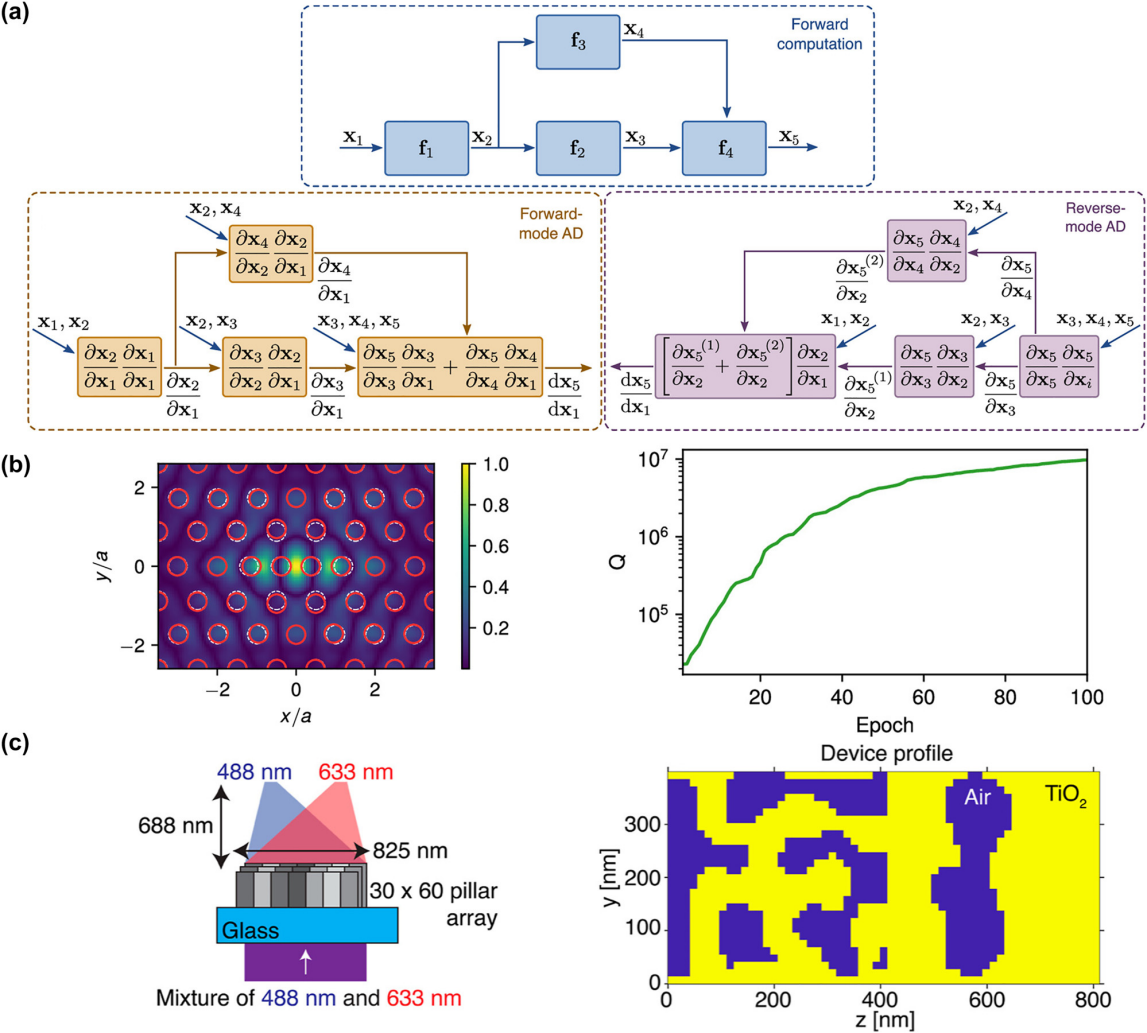
Examples and applications in inverse design of automatic differentiation (AD) and direct differentiation (DD). (a) Computational graphs of forward-mode AD and reverse-mode AD in a fan-in and fan-out computation example. Adapted with permission from Ref. [59]. Copyright 2020 American Chemical Society. (b) Optimized high-quality factor (*Q*) cavity (left) and a graph of *Q* versus epoch (right). White dashed lines in the left figure indicate the position of the initial structures. Reprinted with permission from Ref. [59]. Copyright 2023 American Chemical Society. (c) Illustration of inverse-designed color sorter using DD in the frequency domain, depicting a schematic (left) and optimized permittivity distribution (right) for spatial separation of two different wavelengths (488 nm, 633 nm). Reprinted with permission from Ref. [162]. Copyright 2023 American Chemical Society.

In this context, automatic differentiation (AD) presents itself as an alternative for obtaining the gradient of intricate merit functions [178]. AD uses the chain rule to calculate the gradient, which is similar to the backpropagation process of deep learning. With this method, the gradient is directly calculated by leveraging AD libraries such as JAX [179] and Autograd [180], thereby removing the necessity for manual derivation. AD operates through two distinct modes: forward and reverse. The theoretical detail of applying both modes to inverse design was described by Hughes et al. [161], Minkov et al. [59], and Tang et al. [162]. Forward-mode AD computes the gradient in forward order by accumulating the Jacobian with the chain rule. In contrast, in reverse-mode AD, the gradient is computed in the opposite direction. For the detailed expression, suppose a computation system illustrated in Figure 8a, where

(8) $$\begin{array}{ll} x_{2} & =f_{1}\left(x_{1}\right),\\ x_{3} & =f_{2}\left(x_{2}\right),\\ x_{4} & =f_{3}\left(x_{3}\right),\\ x_{5} & =f_{4}\left(x_{3},x_{4}\right). \end{array}$$

The Jacobian of this fan-in and fan-out system can be represented as

(9) $$\frac{\text{d}x_{5}}{\text{d}x_{1}}=\left[\frac{\partial x_{5}}{\partial x_{4}}\frac{\partial x_{4}}{\partial x_{2}}+\frac{\partial x_{5}}{\partial x_{3}}\frac{\partial x_{3}}{\partial x_{2}}\right]\frac{\partial x_{2}}{\partial x_{1}}.$$

The forward-mode derivative computation accumulates the Jacobian through the addition of derivatives, which corresponds to the brown box in Figure 8a. The primitive computing block of the forward-mode calculation is

(10) $$G_{\text{FM}}\left(x_{i},x_{j},\frac{\partial x_{j}}{\partial x_{1}}\right)=\frac{\partial x_{i}}{\partial x_{j}}\frac{\partial x_{j}}{\partial x_{1}}$$

where **x**_**i**_ indicates the input from the forward-mode computation. Since the forward-mode computes the gradient with **x**_**1**_ which is an input parameter of the system, the computation time linearly scales depending on the number of inputs. On the other hand, a method that accumulates the Jacobian in the opposite direction is called reverse mode, shown in the purple box of Figure 8a. The primitive computing block of the reverse-mode calculation can be represented as

(11) $$G_{\text{RM}}\left(x_{i},x_{j},\frac{\partial x_{5}}{\partial x_{i}}\right)=\frac{\partial x_{5}}{\partial x_{i}}\frac{\partial x_{i}}{\partial x_{j}}.$$

One main difference between forward-mode AD and reverse-mode AD arises from these operations of primitive blocks. Unlike forward mode computation, the time complexity of the reverse mode linearly scales with the number of outputs. The memory complexity also differs between the two modes. In forward-mode AD, the derivatives can be computed in parallel with the forward computation, thus obviating the necessity to store the results of individual steps. Conversely, reverse-mode AD requires the storage of all intermediate values because it computes the gradient after the forward computation has concluded [59].

Tang et al. [162] compared time and memory scaling complexities of finite-difference method, adjoint method, forward-mode AD, reverse-mode AD, and their own method, direct differentiation. The complexities were calculated based on adapting those manners in FDTD simulation. As shown in Table 1 of their work [162], the time complexities of the adjoint method and reverse-mode AD are both represented as *O*(*N*_output_ *N*_*V*_ *N*_*T*_ *N*_*f*_), whereas forward-mode AD’s time complexity is represented as *O*(*N*_input_ *N*_*v*_ *N*_*T*_ *N*_*f*_). Here, *N*_*V*_ is the number of spatial grid pixels, *N*_*T*_ is the number of time steps in simulation, and *N*_*f*_ denotes the number of frequency points. Since the forward-mode AD’s computation time depends on the number of inputs (*N*_input_), from the perspective of computational time the adjoint method and reverse-mode AD can be more expedient in scenarios involving a greater number of input variables (*N*_input_ ≫ *N*_output_) [178], [181].

In terms of memory consumption, on the other hand, the adjoint method and forward-mode AD have the same complexity with *O*(*N*_input_ *N*_*V*_ *N*_*f*_). In contrast, the memory complexity for reverse-mode AD is represented as *O*(*N*_output_ *N*_*V*_ *N*_*T*_ *N*_*f*_) which linearly scales with *N*_*T*_ and *N*_output_. Therefore, in designing large-scale devices, one needs to take into consideration the number of input and output parameters as well as the simulation time steps of the problem to select the method that is suitable for the memory and time constraints.

Hughes et al. [161] implemented forward mode AD into the FDTD simulation which has benefits in solving problems which involves several desired characteristics that need to be determined (*N*_output_). They used forward-mode AD to calculate the accurate gradient of the electric field intensity distribution. Minkov et al. [59] utilized reverse-mode AD and FDTD simulation to optimize the dispersion of a photonic crystal waveguide and to improve the quality factor (*Q*) of a photonic crystal cavity. Since one needs to solve eigenvalue problems to design the photonic crystal, it is theoretically challenging to calculate the gradient with the adjoint method. They used the AD package called Autograd [180] and implemented the plane-wave expansion and the guided-mode expansion. As shown in Figure 8b, they optimized a lithium niobate (LN) photonic crystal cavity to have higher *Q* and lower mode volume by calculating the gradient with AD.

The application of AD method was not limited to FDTD simulation. Su et al. [182] designed three-dimensional (3D) wavelength demultiplexers utilizing FDFD simulation and reverse mode AD. They introduced a nanophotonic inverse design framework called SPINS [183], which is gradient-based optimization. In addition, Shane et al. [138] adopted reverse-mode AD in RCWA since RCWA has advantages in designing periodic structures, which is the case of various applications in meta-optics. They also implemented a parameterization method with the reverse mode AD-based topology optimization and achieved highly efficient metagratings and metasurfaces.

Recently, although it is slightly out of the scope of this chapter, Tang et al. [162] introduced a gradient computation method called direct differentiation (DD) which is specialized to the FDTD computational tree. As they described the mathematical details in their paper, DD is a method to analytically differentiate the mathematical update-equations and propagate the gradients of FoM in reverse direction FDTD simulation. Since DD stores the fields at the last time step, it has relatively low memory complexity compared to the reverse-mode architecture which stores every field data during forward pass. They represented the time complexity of DD as *O*(*N*_output_ *N*_*V*_ *N*_*T*_ *N*_*f*_) and memory complexity as *O*(*N*_output_ *N*_*V*_ *N*_*f*_ + *N*_*T*_ *N*_*V*_∂*N*_*V*_). They utilized DD to optimize a resonant nanostructure array and a color sorter that splits the light depending on the frequency as illustrated in Figure 8c.

### 4.3 Neural network-based approaches

Optimization methods using neural networks have flourished owing the synergy between the advancements in GPU computing power and the timely integration of artificial intelligence [36], [184]. This synergy has propelled the use of machine learning to enhance design processes and addressing complex spectral features, such as multiple resonances and dual polarization in photonic structures. Consequently, these approaches have emerged as end-to-end design methodologies, offering comprehensive design solutions that transcend the constraints of conventional forward and inverse design approaches, thereby marking a pivotal progression in photonics.

Deterministic models in the field of photonics, particularly for neural network-based sections, employ a predefined set of rules to predict outcomes with certainty, given a specific set of initial conditions. Unlike probabilistic models that incorporate randomness, deterministic models provide accurate predictions of the light behavior within the designed systems. Their precision is indispensable in applications where exact solutions are necessary, such as the design and simulation of photonic devices, including waveguides, resonators, and photonic crystals. Within neural networks, deterministic modeling may leverage deep learning to map input parameters such as geometric configurations to output responses such as transmission spectra, thereby streamlining the design process by sidestepping traditional, resource-intensive simulations. This approach can significantly accelerate the design process by bypassing the traditional computationally intensive electromagnetic simulations. For example, a neural network trained on a dataset of simulation or experimental results can learn the relationship between design parameters and optical properties, enabling the quick prediction of new designs.

For more complex design challenges, alternative approaches that move beyond the limitations of predefined rules are being increasingly explored to address more complex design challenges. This has led to the development of generative models, a class of machine learning models trained to understand the underlying data distributions to produce new, synthetic instances of data. Initially rooted in statistical methodologies, they have evolved to incorporate advancements in deep learning, diversifying significantly throughout their development. Variational autoencoders (VAEs) and generative adversarial networks (GANs) are two prominent examples with increasing applications in photonic design research [185]–[187]. Moreover, the diffusion process, which has recently gained traction in the image generation domain, is also being explored for integration into current research endeavors.

However, both deterministic and generative models depend heavily on the quality of training data. While generative models offer innovative solutions, they also face challenges such as instability during training and the complexities of generating large volumes of high-quality data, making the design of large-area structures particularly challenging.

To address these limitations and explore alternative training methods involves ongoing exploration of the potential of physics-informed neural networks (PINNs). This approach integrates physical equations directly into the neural network architecture, allowing the network to learn from both data and physical principles. Jiang et al.’s [188] study introduces a novel methodology that utilizes conditional generative neural networks for the global optimization of dielectric metasurfaces. Unlike conventional optimization techniques, this approach does not depend on predefined datasets for training. Instead, it incorporates a physics-driven mechanism that directly utilizes electromagnetic simulations to refine and optimize the distribution of devices towards achieving high efficiency [189]–[191]. This innovative technique significantly enhances computational efficiency in designing metagratings, achieving results comparable or superior to adjoint-based topology optimization methods, but with considerably reduced computational costs. Furthermore, integrating models with PINN allows this approach to be applied to large-scale designs, thereby opening new avenues for efficient and effective optimization.

Parallel to the advancements in generative modeling, research on the application of reinforcement learning (RL) in photonics is progressing rapidly, offering a new perspective for optimizing photonic devices. Seo et al. [63] have made strides in this area by introducing a deep reinforcement learning (DRL) strategy that employs a deep *Q*-network (DQN) agent to efficiently navigate and optimize the design space of devices, without requiring prior data. This method significantly outperforms traditional approaches in terms of efficiency across various wavelengths and deflection angles, owing to the ability of RL to handle complex, multidimensional design spaces. Despite its potential, the application of RL to large-scale projects raises concerns regarding the computational intensity of the simulations. However, the potential of RL in the optimization of photonic devices indicates a move towards scalable and effective solutions for the development of high-performance metasurfaces, with the caveat that overcoming computational hurdles is essential for its broader application.

In this review, we have explored the use of deterministic models, generative models, and reinforcement learning-based optimization techniques in photonics design. By applying PINN, we have identified the degree of potential for addressing the challenges of data generation for these designs. However, significant issues still need to be addressed, particularly in applying these methods to large-scale designs owing to data generation constraints and the limitations posed by the GPU memory capacity when handling extensive datasets. In the following section, we aim to investigate the feasibility of applying large-scale optimization by combining neural network-based optimization with gradient-based methods and examining relevant research examples.

### 4.4 Neural network combined with the gradient-based optimization

The fusion of neural networks with topology optimization has sparked a significant wave of innovation in the domain of large-scale nanophotonic design. Highlighting this trend, Gershnabel et al. [192] are at the forefront of a reparameterization strategy for gradient-based inverse design, enabling precise geometric control and thereby setting a benchmark in the domain. Their technique is distinctive in its integration of reparameterization, which enables the precise adjustment of geometric parameters to optimize nanophotonic devices. This approach demonstrates the potential of combining artificial neural networks (ANNs) and topology optimization to efficiently streamline the design of intricate optical devices efficiently. By addressing the high computational demands and geometric constraints of traditional design methods, their study underscores the potential role of machine learning in enhancing and simplifying the design process, potentially paving the way for more advanced nanophotonic applications.

**Figure 9:**
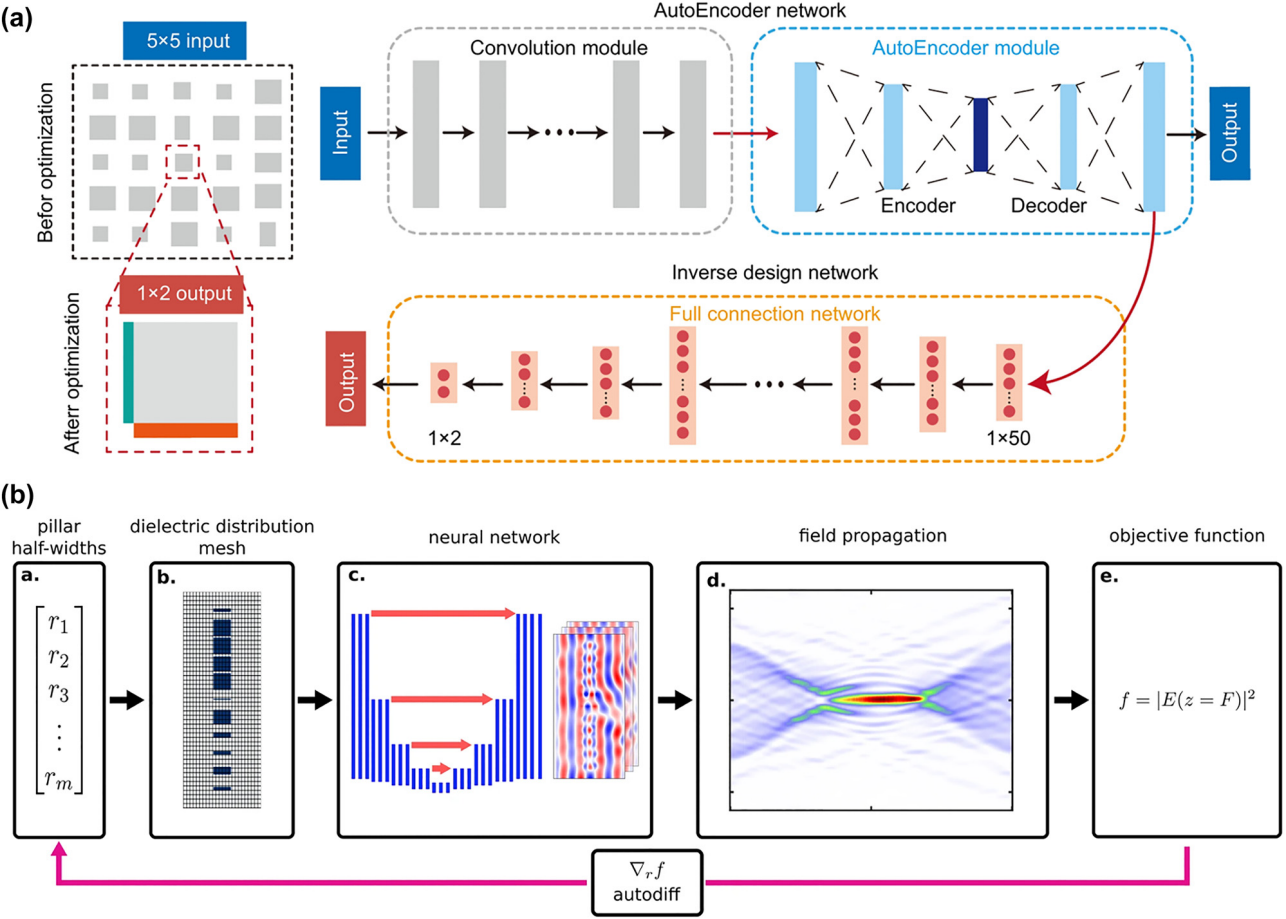
Inverse design framework incorporating neural network and adjoint optimization. (a) The optimized network framework comprises an A-network that enhances the information space of sampled data, while the I-network filters the weak coupling strength structures [193]. Licensed under CC BY 4.0. (b) Optimization strategy of 2D meta-optics with PINN [194]. Licensed under CC BY 4.0.

Figure 9a demonstrates Ha et al.’s [193] physics-data-driven approach, which combines multi-objective optimization and deep learning for a large-aperture metalens design, exemplifying efficiency with notable focusing capabilities. This innovative model employs a synergistic combination of multi-objective optimization algorithms and deep learning to accelerate the design process while achieving remarkable efficiency. Focusing on a large-scale 1 mm diameter metalens, Ha et al. have attained an impressive relative focusing efficiency of 93.4 % and a Strehl ratio of 0.94. Their work represents the successful integration of ANN-based techniques with topology optimization, addressing computational obstacles in the large-scale simulation and the design of sophisticated nanophotonic devices. Further pushing the boundaries to overcome the data generation challenges of designing large-scale photonic structures, Zhelyeznyakov et al. [194] have adopted a data-free machine learning approach employing PINN as illustrated in Figure 9b. This strategy significantly mitigates the need for extensive data and streamlining the design process. The significance of their work lies in demonstrating the potential of efficiently designing large aperture meta-optics with enhanced performance, thereby showcasing the utility of PINN in advancing optical design and computational electromagnetics methodologies. By utilizing neural network-based and gradient-based optimizations for designing large-area photonic structures, has resulted in addressing computational challenges have been addressed. This includes the prohibitive computational cost of full-wave simulations over large domains, which have traditionally presented issues with operational complexity and memory consumption. By significantly reducing the computational burden, these innovative approaches promise to become breakthroughs in large-scale design, transforming the field with more efficient and higher-performance nanophotonic applications. As GPU capabilities evolve alongside deep learning and reinforcement learning technologies, research combining gradient-based optimization with these methods is expected to expand, promising further advancements in this field.

In this section, we examined instances in which traditional neural network-based optimization, when applied on a large scale, encounters expected challenges when applied on a large scale such as large-scale data generation, memory management for extensive datasets, and increased computational demands. We explored how integrating these techniques with gradient-based approaches can mitigate their drawbacks and maximize their strengths in designing large-scale photonic structures. For large-scale design efforts to reduce the computational load, an appropriate electromagnetic solver must be selected, and opt for lower-complexity optimization methods must be chosen. Consequently, future research should focus on developing and applying suitable optimization methods for large-scale applications, which will be pivotal for achieving more extensive design objectives.

## 5 Discussion and outlooks

The Large-scale inverse design of photonic devices has already made significant progress and is actively reshaping the design approach within the field of photonics. However, the realization of the full potential inherent in large-scale design requires progress in algorithmic strategies for inverse design, enhanced speed in computing unit simulations, and the implementation of hardware-accelerated design techniques.

This review presents the latest developments in large-area inverse design, facilitated by hardware acceleration, algorithmic innovations, and alternative methodologies. The primary design approach of metasurfaces, referred to as ‘unit-cell’ design, revolutionized large-area photonic devices. Despite its impact, this strategy faces obstacles in developing devices that are capable of multifunctional or broadband operations, especially because of the challenges associated with designing meta-atoms that accommodate rapidly varying wavefronts across both the spatial and frequency domains. The inverse design introduces a paradigm transition to addressing the limitations of metasurface designs that rely on meta-atoms. This approach applies new mathematical models to navigate physical constraints; however, it often requires numerous iterations of full-scale simulations, presenting a major hurdle for ultra-large-scale optimizations.

In response to the computational challenges in designing ultra-large-scale photonic devices, comprehensive strategies have been introduced via hardware acceleration, deep learning techniques, and algorithmic innovations. Hardware acceleration markedly reduces the simulation time primarily through the emergence of high-performance GPUs and specialized processors, thereby fostering more efficient design exploration and optimization. Concurrently, deep learning models are promising candidates for approximating the solution of Maxwell’s equations with less computational overhead. In addition, pre-trained deep learning models even offer highly efficient photonic designs without any additional simulations.

As a final remark, emerging techniques for large-scale inverse designs are yet to be matured and should be further improved. For example, the collection and normalization of extremely large amounts of simulation data may open a way of training the large Language model (LLM) for electromagnetics. This LLM may understand the rule of Maxwell’s equations and corresponding light matter interaction in many different environments. Scale invariance in electromagnetic theory, the property of the equation remains unchanged under a scale transformation, which may be one example that can significantly reduce amount of training data. In a similar way, we expect to observe different types of breakthroughs in theoretical, numerical, or physical (hardware) solutions. A new computer architecture could be a physical breakthrough such as processing in memory computing [195] and neural processing units. Ultimately, the ongoing advancement in large-scale inverse design techniques is poised to transform the field of photonics, expanding the limits of technological possibilities and pave the way for innovative research and applications.
